# Lost in promiscuity? An evolutionary and biochemical evaluation of HSD10 function in cardiolipin metabolism

**DOI:** 10.1007/s00018-022-04579-6

**Published:** 2022-10-22

**Authors:** Yvonne Wohlfarter, Reiner Eidelpes, Ryan D. Yu, Sabrina Sailer, Jakob Koch, Daniela Karall, Sabine Scholl-Bürgi, Albert Amberger, Hauke S. Hillen, Johannes Zschocke, Markus A. Keller

**Affiliations:** 1 Institute of Human Genetics, Medical University of Innsbruck, Peter-Mayr-Str. 1/1.OG, 6020 Innsbruck, Austria; 2 Department of Medical Biochemistry and Biophysics, Karolinska Institute, Stockholm, Sweden; 3 Department of Cellular Biochemistry, University Medical Center Göttingen, Göttingen, Germany; 4 Research Group Structure and Function of Molecular Machines, Max Planck Institute for Multidisciplinary Sciences, Göttingen, Germany; 5 Department of Paediatrics I (Inherited Metabolic Disorders), Medical University of Innsbruck, Innsbruck, Austria; 6 Cluster of Excellence ‘Multiscale Bioimaging: from Molecular Machines to Networks of Excitable Cells’ (MBExC), University of Göttingen, Göttingen, Germany

**Keywords:** Phospholipase C, Cardiolipin, Tafazzin, Evolution, Enzyme function

## Abstract

Multifunctional proteins are challenging as it can be difficult to confirm pathomechanisms associated with disease-causing genetic variants. The human 17β-hydroxysteroid dehydrogenase 10 (HSD10) is a moonlighting enzyme with at least two structurally and catalytically unrelated functions. HSD10 disease was originally described as a disorder of isoleucine metabolism, but the clinical manifestations were subsequently shown to be linked to impaired mtDNA transcript processing due to deficient function of HSD10 in the mtRNase P complex. A surprisingly large number of other, mostly enzymatic and potentially clinically relevant functions have been attributed to HSD10. Recently, HSD10 was reported to exhibit phospholipase C-like activity towards cardiolipins (CL), important mitochondrial phospholipids. To assess the physiological role of the proposed CL-cleaving function, we studied CL architectures in living cells and patient fibroblasts in different genetic backgrounds and lipid environments using our well-established LC–MS/MS cardiolipidomic pipeline. These experiments revealed no measurable effect on CLs, indicating that HSD10 does not have a physiologically relevant function towards CL metabolism. Evolutionary constraints could explain the broad range of reported substrates for HSD10 in vitro. The combination of an essential structural with a non-essential enzymatic function in the same protein could direct the evolutionary trajectory towards improvement of the former, thereby increasing the flexibility of the binding pocket, which is consistent with the results presented here.

**Supplementary Information:**

The online version contains supplementary material available at 10.1007/s00018-022-04579-6.

## Introduction

Human 17β-Hydroxysteroid dehydrogenase 10 (HSD10; EC 1.1.1.3; OMIM 3000256) is a multifunctional mitochondrial enzyme encoded by the *HSD17B10* gene on chromosome Xp11.2 [1–3]. The gene consists of six exons, of which exon 1 to 3 encode the NAD-binding region and exon 4 to 6 encode the catalytic residues of the active site. Pathogenic variants in the gene can cause HSD10 disease, a progressive multi-system disease arising from mitochondrial dysfunction mostly in males leading to clinical presentation patterns from severe infantile to mild juvenile forms [4].

**Fig. 1:**
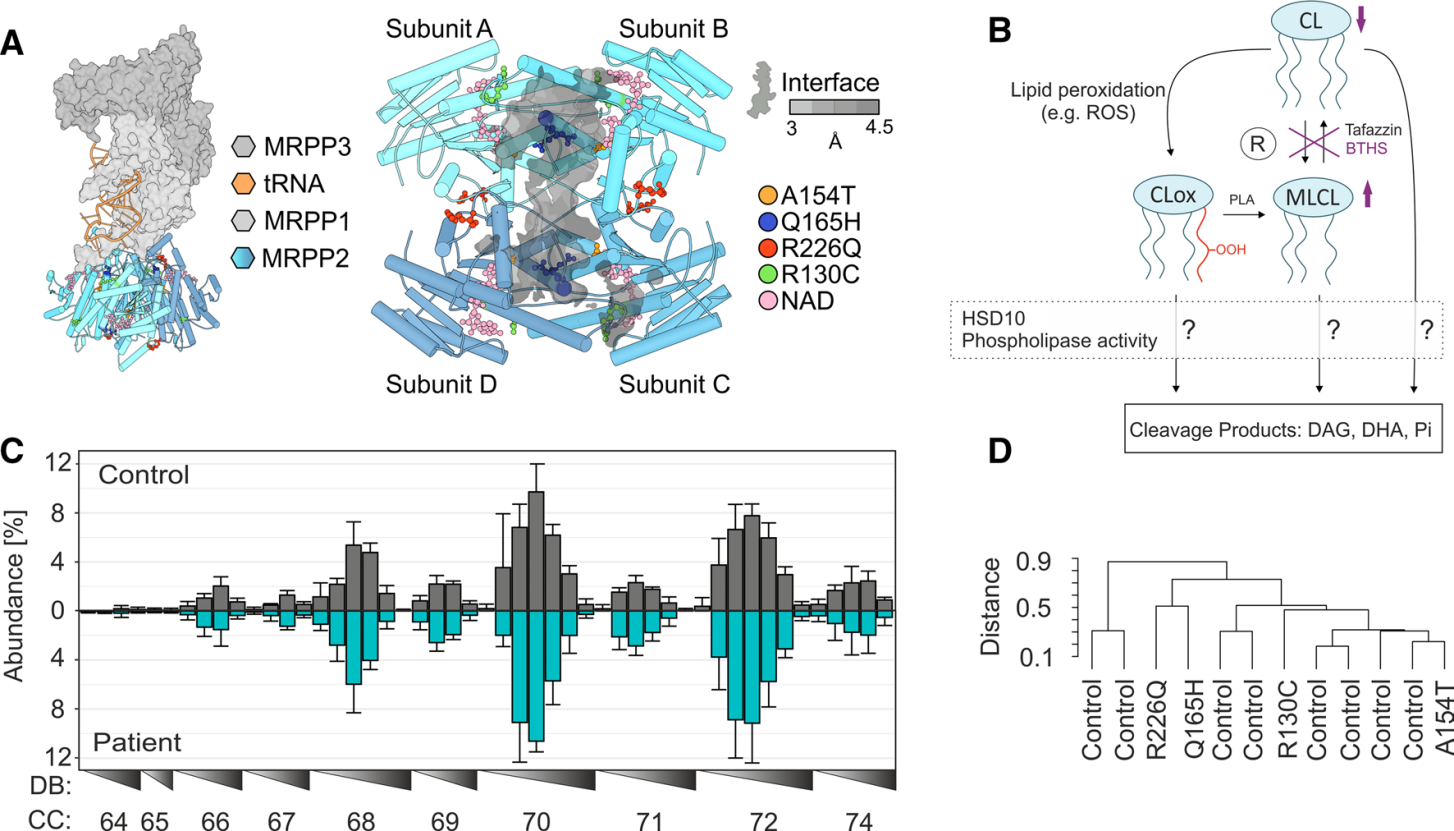
HSD10 structure and effect of proposed phospholipase C-like activity on CL profiles in control and HSD10 disease derived fibroblasts. **A** Left: visualization/depiction of the mtRNase P complex structure, which is made up of MRPP1, MRPP3 and the HSD10 tetramer, including a bound tRNA molecule found in the Protein Data Bank (PDB) under accession code 7ONU [40]. Right: top view of the HSD10 tetramer with patient mutations present in fibroblast cell lines used in this study (Supplemental Table 2) [8, 9, 36, 37]. Cofactor NAD is depicted in yellow). **B** Scheme depicting the possible contribution of the proposed HSD10 phospholipase C-like function of HSD10 to CL homeostasis [11]. HSD10 could impact on the CL constitution on the level of CL, MLCL, as well as CL_ox_. The effects of tafazzin deficiency in Barth Syndrome are indicated in purple. C) Relative abundances of individual CL species in control fibroblasts (Control; *n* = 8) and fibroblasts derived from HSD10-disease patients (Patient; *n* = 4). Profiles are shown as mean ± SD and are normalized to total CL. CL species are indicated by their total number of side chain carbon atoms (CC) and sorted according to the number of double bonds (DB). Individual CL profiles are shown in Supplemental Figure S1 and S2. One-way analysis of variance showed that the CL profiles of patient fibroblasts were not significantly different from control fibroblasts (*F*(1,586) = 0, *p* = 1). **D** Complete-linkage hierarchical clustering results of CL patterns depicted as dendrogram. Identical data as shown in **C** were used. The underlying concordance matrix was constructed using the Kendall method

In its core function, HSD10 (also denoted MRPP2) serves as an essential component of the mitochondrial mtRNase P complex, in which it forms an RNA-binding platform together with MRPP1 (Fig. 1A). mtRNase P is located in proximity of the inner mitochondrial membrane (IMM) and represents the first enzyme involved in the processing of the polycistronic mtDNA transcript, cleaving the 5’ end of mitochondrial precursor tRNAs [5–7]. Deficiency of this function is thought to cause the clinical presentation of HSD10 disease [8]. HSD10 serves a completely different function as a homotetramer in the mitochondrial matrix where it acts as a dehydrogenase that converts the isoleucine catabolite 2-methyl-3-hydroxybutyryl-CoA into its product 2-methylacetoacetyl-CoA. Additionally, HSD10 has been described to have many different moonlighting enzymatic activities in various metabolic pathways (see Supplemental Table 1) [9–20].

These reactivities were predominantly identified on basis of in vitro studies. Potential physiological substrates include fatty acids, sex hormones, and steroids. However, the actual physiological relevance of these functions is so far uncertain. As a consequence of this heterogeneity, HSD10 has been described with a series of different alternative names such as MHBD, ABAD, MRPP2, SCHAD, ERAB, SDR5C1, HADH-II, and others, sometimes leading to confusions in research literature.

All pathogenic *HSD17B10* variants reported so far at least partially impair the enzymatic function of HSD10. Consequently, the disease is often recognized by excretion of 2-methyl-3-hydroxybutyrate in the patients’ urine [4, 9]. However, the severity of symptoms is independent from the level of residual dehydrogenase enzyme activity. Thus, it has been concluded that pathogenesis in HSD10 disease is independent from the protein’s dehydrogenase function but is instead caused by impaired assembly of the mtRNase P complex required for mtDNA transcript processing [4, 8]. The concept that a complete/permanent loss of the HSD10 protein is incompatible with life [4] is supported by the absence of null (stop/splice/frameshift) variants in population databases.

Recently, it has been suggested that HSD10 exhibits a specific phospholipase C-like activity towards CLs, cleaving them into diacylglycerol (DAG), dihydroxyacetone (DHA) and orthophosphates (Pi) as reaction products [11]. CLs are dimeric, glycerol-bridged phospholipids that are predominantly found in the inner mitochondrial membrane and are required for the formation of cristae and for supporting the (super-) complex assembly [21, 22]. The CL composition of a cell is maintained by its initial biosynthesis [23–26], a remodeling process that optimizes the fatty acyl side chain constituent/substation of CLs and is largely mediated by the transacylase tafazzin [27, 28], and its degradation [25, 28]. Mutations in the *TAFAZZIN* gene lead to the severe x-linked disorder Barth Syndrome, which is characterized by cardiomyopathy, muscle weakness, neutropenia, and 3-Methylglutaconic aciduria [29–31]. The described phospholipase C-like activity of HSD10 has been reported to accelerate in in vitro assays with increasing polarity of the CL substrate, causing a preference for oxidized CLs (CL_ox_). Mitochondria are a main cellular source for reactive oxygen species (ROS) [32], which (among other factors) are responsible for the oxidation of CLs into CL_ox_. It has been hypothesized that the HSD10-dependent breakdown of CL_ox_ species prevents their translocation to the outer mitochondrial membrane, which would otherwise trigger an apoptotic signalling cascade [21, 32].

In this study, we characterize the lipidomic consequences of the proposed CL-cleaving activity of HSD10 in living cells and patient fibroblasts. This represents an important step in evaluating whether it was justified to grant this specific HSD10 function the central place in CL metabolism, which it has been recently attributed [33–35] on basis of the initial in vitro results [11].

## Results

There are different mechanisms by which a potential CL-degrading function of HSD10 could affect CL homeostasis. Purified HSD10 is able to cleave CLs [11] and has been reported to have an even higher affinity for more polar CL species, which for example can lead to an accelerated breakdown of monolyso-cardiolipins (MLCL) and CL_ox_ (Fig. 1B). This is in line with the reported preference of HSD10 for CL(18:2)_4_ over CL(14:0)_4_ [11]. If these principles hold true in a physiologically relevant cellular context, it can be hypothesized that a lack of the phospholipase C-like activity of HSD10 would result in alterations of CL homeostasis, including the molecular composition of CL species as well as the relative abundances of CL, MLCL, or CL_ox_ (Fig. 1B). In this regard, the strength of an observable HSD10-dependent effect should considerably rely on the underlying CL profiles, the concentrations of MLCL, and the presence of an oxidative environment that leads to increased mitochondrial lipid peroxidation.

### CL compositions of HSD10 disease patient fibroblasts

To test the hypothesis, we first compared the CL compositions of fibroblasts obtained from four different HSD10-deficient patients (Patient), corresponding to four different pathogenic variants in the *HSD10B17* gene (see Supplemental Table 2 and Fig. 1A), with fibroblasts from eight unaffected, healthy controls (Control). All patient cell lines were previously confirmed to be functionally impaired on the basis of HSD10 disease-causing variants [8, 9, 36, 37]. The four different patient variants represent different functional and clinical presentation patterns: variants p.R226Q and p.N247A cause the neonatal presentation and appear to affect tetramerization, while p.R130C and p.P210S, leading to the infantile form, are still able to form a tetramer [7]. Fibroblasts were cultured and CL composition was analyzed by means of HPLC–MS/MS as reported earlier [38, 39]. Figure 1C (for individual CL profiles of patients see Supplementary Figure S1) summarizes the average abundances and diversity of CLs in Patient and Control fibroblasts, for which we could not detect any significant differences. In contrast, when analyzed with the same methodology, Barth Syndrome patient-derived fibroblasts exhibit strongly altered CL compositions [38]. Also, a clustering analysis of all samples according to their CL profiles (hierarchical clustering on basis of Kendall concordance matrix) revealed absence of discriminatory factors and similarity between Patient (indicated by pathogenic variant) and Control fibroblasts (Fig. 1D). Indeed, the cell-line-to-cell-line variability was clearly larger than any potential phospholipase C-like activity of HSD10. Thus, these experiments provided no evidence that the enzymatic function of HSD10 is involved in CL homeostasis in this model system.

### HSD10 knockdown in HEK 293 T cells and its influence on CL homeostasis

**Fig. 2:**
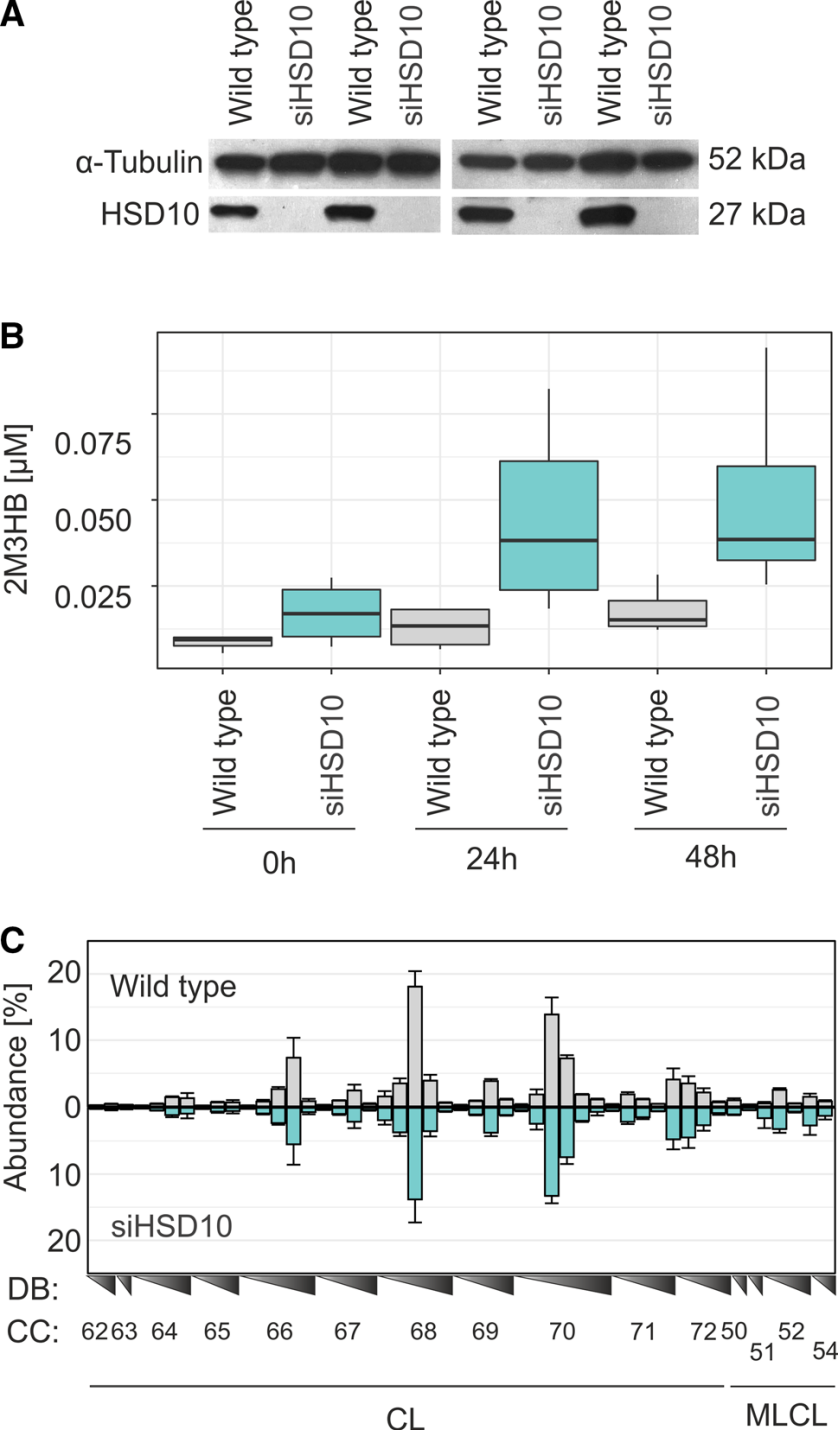
Influence of HSD10 enzyme activity to isoleucine catabolism and CL metabolism in wild type HEK 293 T cells. **A** Confirmation of HSD10 knockdown (siHSD10; *n* = 4) in HEK 293 T cells via western blot analysis, α-Tubulin was used as loading control. HSD10 was detected with the ERAB antibody (ab137455). Silencing of the *HSD17B10* gene with siRNAs led to a strongly diminished signal at 27 kDa, compared to negative control siRNAs (Wild type; *n* = 4). **B** Measurement of 2-methyl-3-hydroxybutyrate (2M3HB) in cell culture medium of HSD10 knockdown in HEK 293 T cells (siHSD10, *n* = 3) and respective HEK 293 T controls (Wild type, *n* = 4). 2M3HB was determined by GC–MS after 0 h, 24 h, and 48 h after challenging cells with 1.36 mM of isoleucine. Boxplots depict the median, interquantile range, as well as the 10- and 90-percentile of each condition. In siHSD10 cells a pronounced accumulation of 2M3HB was observed. As data were not normally distributed (Shapiro–Wilk test showed a significant departure from normality, *p* value = 1.00 × 10^–5^) the non-parametric Kruskal–Wallis test was employed and revealed significant differences between siHSD10 knockdown and Wild type cells (*χ*^2^ = 7.68, *p* = 0.005584). **C** Abundance of CL species in Wild type and knockdown siHSD10 cells. CL species are indicated by their side chain carbon atoms (CC) and sorted according to the number of double bonds (DB) in analogy to Fig. 1C. Data are shown as mean ± SD (Control *n* = 3, siHSD10 *n* = 4). A one-way analysis of variance showed the absence of any significant effect of the HSD10 knockdown (*F*(1,453) = 0, *p* = 1)

As the contribution of HSD10 to CL homeostasis could be potentially masked by the choice of model system and growth environment, we next established a HSD10-deficient cell culture model in HEK 293 T cells. This was achieved by silencing *HSD17B10* expression with siRNAs (siHSD10), with non-silencing siRNAs serving as controls (Wild type). Western blot analysis confirmed an almost complete loss of the HSD10 protein, compared to controls (Fig. 2A). The loss of the enzymatic activity of HSD10 was verified by determining 2-methyl-3-hydroxybutyrate concentrations in an isoleucine enriched cell culture medium after 0 h, 24 h and 48 h by means of gas-chromatographic-mass spectrometric (GC–MS) measurements. In line with the patients’ phenotypes [4], we observed that HSD10-deficient cells accumulated 2-methyl-3-hydroxybutyrate compared to their respective controls (Fig. 2B). Silencing *HSD17B10* expression was strong enough to diminish this core enzymatic activity, allowing to also investigate its potential CL-cleaving function. Using these cells, we next characterized the CL composition in siHSD10 and wild type cells using our established HPLC–MS/MS approach (Fig. 2C). In analogy to the situation in fibroblasts, we again observed no significant changes in the abundance and/or composition of individual CL species in response to the modulation of HSD10 enzyme levels (Fig. 2C). Thus, these results as well did not provide any supporting evidence for a potential physiological contribution of the proposed phospholipase C-like activity of HSD10 [11] to CL homeostasis.

### HSD10 knockdown in tafazzin-deficient HEK 293 T cells and its influence on CL homeostasis

**Fig. 3:**
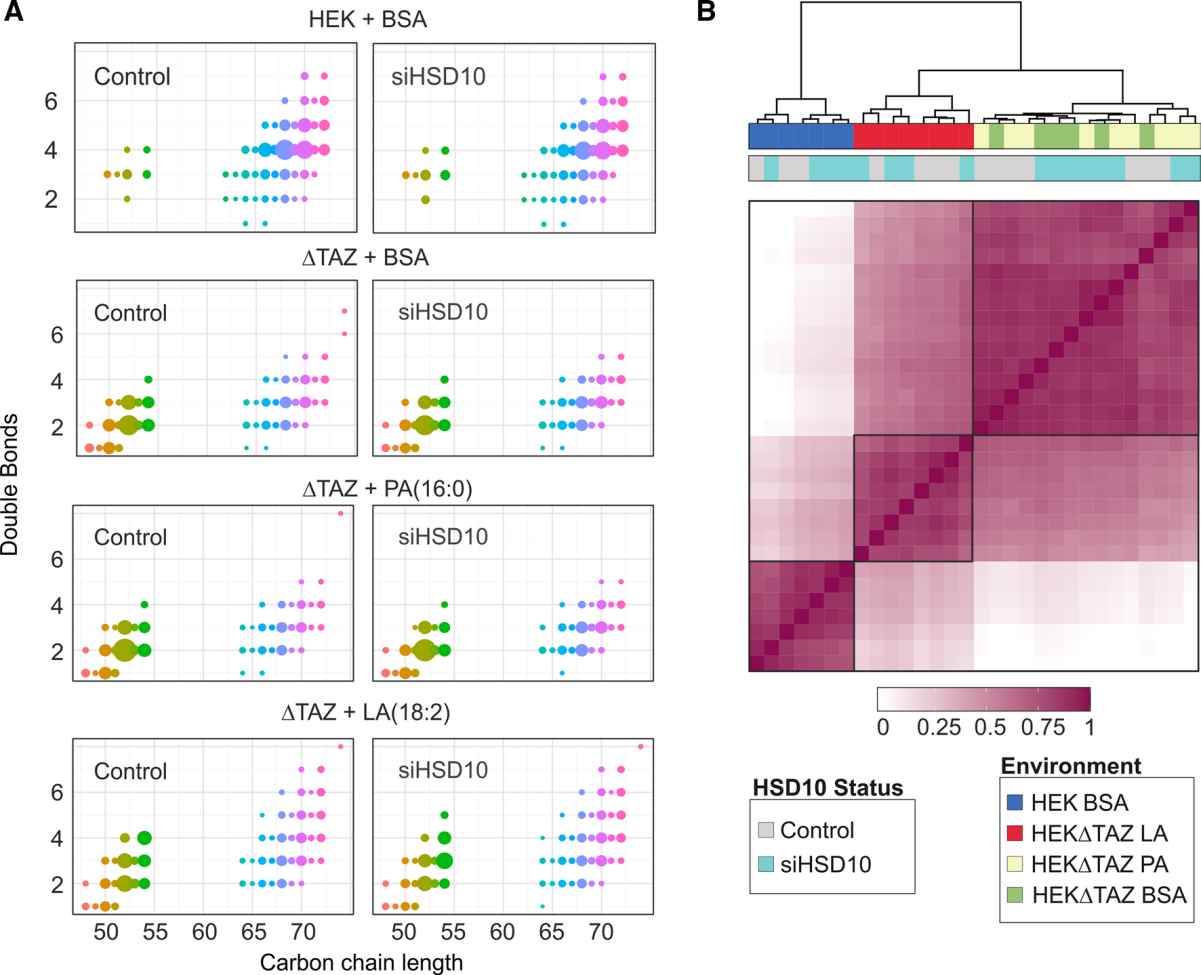
Influence of HSD10 enzyme activity modulation in tafazzin-deficient HEK 293 T cells. **A** Relative single CL profiles with their respective carbon chain length and double bond amount, whereas the dot size corresponds to the relative abundance of CLs. Plot was normalized to 1, which represents the total amount of CLs. Comparison between control cells (Controls) and HSD10 knockdown cells (siHSD10) wild type HEK 293 T cells (HEK) and tafazzin-deficient HEK 293 T cells (∆TAZ). HEK + BSA Data are shown in detail in Fig. 1C and added here for better comparison between wild type and tafazzin-deficient cells. Cells were supplemented with linoleic (LA (18:2)) and palmitic acid (PA (16:0)) to create a polyunsaturated and saturated environment. A one-way analysis of variance with post hoc correction showed that the effect of HSD10 knockdown was not significant (*F*(5,1489) = 0, *p* = 1). **B** Heatmap depicting the Kendall-based concordance matrix between individual CL states of samples shown in **A** and Fig. 2B. Clustering and the resultant dendrogram were constructed by complete-linkage hierarchical clustering. These results suggest a high similarity within each cluster of genetic background (Controls (HEK) vs. tafazzin deficient (∆TAZ)) and altered lipid environment irrespective of the presence or absence of HSD10 (turquoise and gray)

A substrate preference for less hydrophobic CL species has been described for HSD10 in vitro [11]. Thus, we continued by establishing HSD10 knockdowns in the background of tafazzin-deficient HEK 293 T cells (∆TAZ) (western blot verification is seen in Supplementary Figure S3). Tafazzin deficient cells characteristically accumulate more polar MLCL species and exhibit an enhanced production of ROS, which are the major cause for non-enzymatic oxidation events in cells [41, 42]. Especially polyunsaturated CL species, which make up 80% of all species in high energy-demanding tissues such as the heart, are prone to (per)oxidation [43, 44]. Thus, to mimic the physiological polyunsaturated state of CLs, we supplemented the cells with LA (18:2), and used PA (16:0) as saturated fatty acid control. HPLC–MS/MS analysis of tafazzin-deficient cells with modulated lipid availability and modulated HSD10 enzyme activity showed that administration of LA clearly increased the occurrence of polyunsaturated lipids (Fig. 2A). Additionally, tafazzin-deficient cells showed accumulation of MLCLs and reduced complexity of CL patterns due to preferential depletion of polyunsaturated fatty acyl-rich CLs, which is in line with previous observations [38]. However, HSD10 knockdown had no relevant effect on the abundance and molecular compositions of CL species in any of the tested conditions (see Fig. 3A and Supplementary Figure S4). This was also supported by hierarchical clustering analysis using the complete-linkage method and applying the Kendall’s tau for comparison of the various CL profiles (Fig. 3B). While we observed strong effects of the presence or absence of tafazzin, and in response to the lipid environment provided via the medium (blue, red, light yellow, and green), samples remained almost indistinguishable independent of whether HSD10 function was silenced or not (grey and turquoise). As a consequence, also here we did not observe any evidence whatsoever that would support a physiological CL-cleaving function of HSD10 in living cells.

### Biochemical assessment of HSD10 enzyme activity

**Fig. 4:**
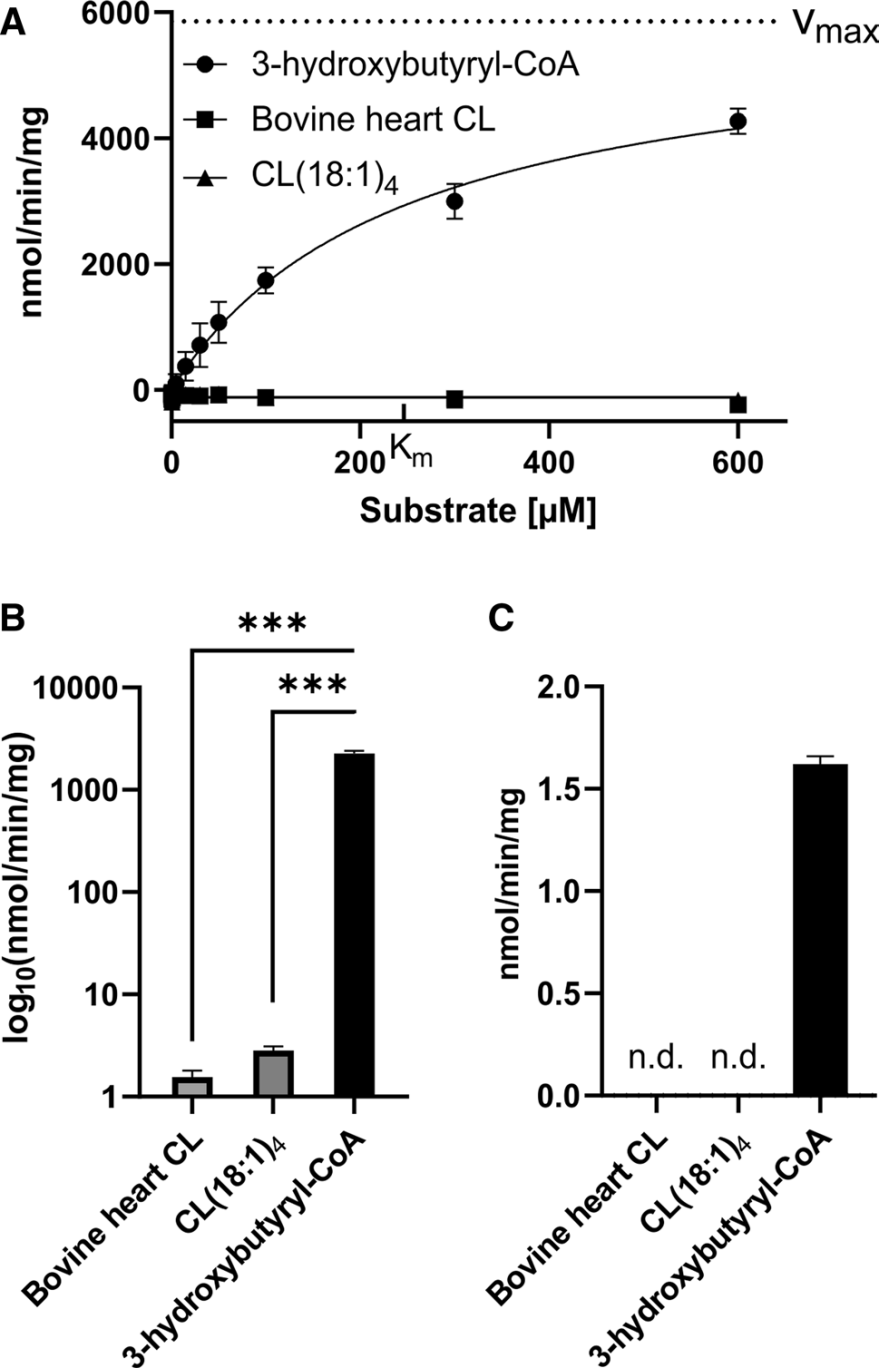
HSD10 enzymatic activity. **A** Michaelis–Menten kinetics of purified HSD10 using 3-hydroxybutyryl-CoA (black circle), Bovine heart CL (black square) and CL(18:1)_4_ (black triangle) as substrates. No activity could be measured using CLs as substrates. With 3-hydroxybutyryl-CoA *V*_max_ = 5857 nmol/min/mg and *K*_m_ = 246.1 µM were determined (*n* = 3). B) Purified HSD10 activity in nmol/min/mg measured with 300 µM of each substrate, respectively (*n* = 3). For both CL substrates, 6 µg HSD10 were used to observe measurable activity, while 3-hydroxybutyryl-CoA was assayed with 60 ng HSD10. **C** HSD10 activity of HEK 293 T cells in nmol/min/mg. No activity towards CL could be detected (n.d.). Data are displayed as mean ± SD. ***p < 0.0001

As it was not possible to detect any significant effect of HSD10 on CLs in various experiments performed with living cells, we next conducted enzyme activity assays using recombinantly expressed and purified HSD10 protein. A fluorometric assay monitoring NADH production was used to determine the enzymatic activity. HSD10 was highly active when assayed with 3-hydroxybutyryl-CoA as substrate, and responded in a Michaelis–Menten-like manner to substrate concentrations ranging from 0 to 600 µM (Fig. 4A, full circle). In contrast, with the same assay conditions we observed no detectable NADH formation (detection limit: 84 pmol NADH) when tetraoleoylcardiolipin as well as a bovine heart CL extract (predominantly tetralinoleylcardiolipin) were used as substrates (Fig. 4A, full square and triangle). A measureable formation of NADH in a CL-dependent manner could only be provoked when increasing the HSD10 protein concentration by two orders of magnitude (60 ng vs. 6 µg). Under these conditions, specific enzymatic activities of HSD10 for both CL substrates were approximately 125-fold smaller when compared with 3-hydroxybutyryl-CoA (Fig. 4B). Using the same measurement principle, we assessed HEK 293 T cell lysates and found no detectable NAD-dependent CL-cleaving reactions, while the positive controls with 3-hydroxybutyryl-CoA as substrates were highly active (Fig. 4C). In summary, these enzyme assay results substantiated our previous observations in living cells and demonstrate that HSD10s cleavage capacity of CLs is dwarfed by its predominant metabolic role.

## Discussion

In the present study we investigated the lipid compositional consequences of the proposed CL-cleaving function of HSD10 that has been previously observed in vitro [11]. Since it was first described, this presumed enzymatic function has already found its way into different representations of the CL metabolic pathways [33–35]. However, an experimental examination of its real physiological relevance had so far been missing. To investigate whether HSD10 indeed is able to cleave CLs in the context of living cells, we analysed the effect of HSD10 on CL patterns across saturated, polyunsaturated and damaged CL species. As varying the lipid availability represents a valuable tool to uncover otherwise masked lipid phenotypes [45, 46], we were additionally establishing different lipid environments in cells with different genetic backgrounds. However, our experiments did not detect any effect of HSD10 on CL composition (Figs. 1C, 2C, 3A, B). In contrast, we could demonstrate the presence and modulation of the dehydrogenase activity of HSD10 in the isoleucine degradation pathway (Fig. 2B), which is used as a diagnostic marker of HSD10 disease [4]. On basis of these data, a physiological contribution of HSD10 to CL homeostasis appears improbable.

In the light of the in vitro kinetic properties of the CL-cleaving function of HSD10 [11], one of the cellular environments in which this function could theoretically be of high relevance is in the context of tafazzin deficiency. The impairment of CL remodeling [24, 27], as well as the more oxidative environment caused by a disruption of (super-)complex assembly [21, 22] and the resulting electron leakage [47], favour the formation of polar CL species which should have an increased binding affinity towards HSD10. However, we again found no differences in the CL states of the respective cells. The comparison of the enzymatic activities of purified HSD10 for the substrates 3-hydroxybutyryl-CoA and CLs, respectively, revealed that the latter is approximately 1400-fold slower and only observable in the presence of high amounts of purified enzyme (Fig. 4). Analogous results have been reported in other publications when comparing the turnover numbers of HSD10 for 3-hydroxybutyryl-CoA with other proposed substrates [6]. Thus, HSD10 facilitated CL cleavage represents a slow side reaction with questionable relevance for the overall metabolic network. Indeed, whole HEK 293 T cell lysates showed no NAD-dependent CL metabolizing activity (Fig. 4C). It is thus highly advisable not to over-interpret the in vitro phospholipase C-like function of HSD10 until a corresponding physiologically relevant example has been discovered. Even if such an instance is described, it will be important to realistically limit its actual radius of action.

A further factor has to be considered in regard to the reported CL-species dependent substrate specificity of HSD10. CLs are highly abundant in the IMM, but accumulation of small amounts of CL_ox_ activate apoptotic signalling cascades [21]. Thus under physiological conditions, the concentrations of CLs will be much higher compared to CL_ox_. Estimated on basis of CL_ox_ concentrations found in murine heart [48], the maximum physiological mitochondrial CL/CL_ox_ ratio is 55:1, with concentrations of 45 µM for CL and 0.82 µM for CL_ox_. When solved in this scenario for the Michaelis-Menten parameters described in [11], HSD10 would cleave CL and CL_ox_ with reaction velocities of 4.38 µM/min and 0.57 µM/min, respectively. In other words, this enzyme function would cleave CL molecules 7.6-times faster than CL_ox_, reversing the implication of the original statement in regard to the reaction velocity of HSD10 [11]. Nevertheless, even with the uncertainty of the actual protein concentration of HSD10 in mitochondria, the reported reaction velocities would be substantial enough to mitigate a measurable influence on CL levels and composition, which, however, could not be observed in this study.

HSD10 is not new to controversy. Originally there was a scientific debate about whether the actual subcellular localization of HSD10 is in the endoplasmic reticulum or in the mitochondria [12]. The presence of a mitochondrial targeting sequence and a series of carefully conducted experiments were in the end confirmed the latter [14, 49]. Also, the potential enzymatic function as an alcohol dehydrogenase has been controversially discussed [3]. HSD10 has been reported to accept a range of different alcohol substrates including ethanol, isopropanol, octanol, and decanol [10, 19, 20]. However, HSD10 exhibits this activity in enzyme assays with low turnover rates, which raises serious doubts about the physiological relevance of its generalized alcohol dehydrogenase activity [3]. In the present study we also find that the conversion rates of purified HSD10 for CLs are orders of magnitude smaller than for a the substrate that is structurally much closer to the physiological main substrate (Fig. 4). Together with the results in living cells (Fig. 3B), this implies that the biochemical CL-cleaving activity of HSD10 does not play a role in shaping the physiological CL composition even under the extreme condition of a tafazzin deficiency.

**Fig. 5:**
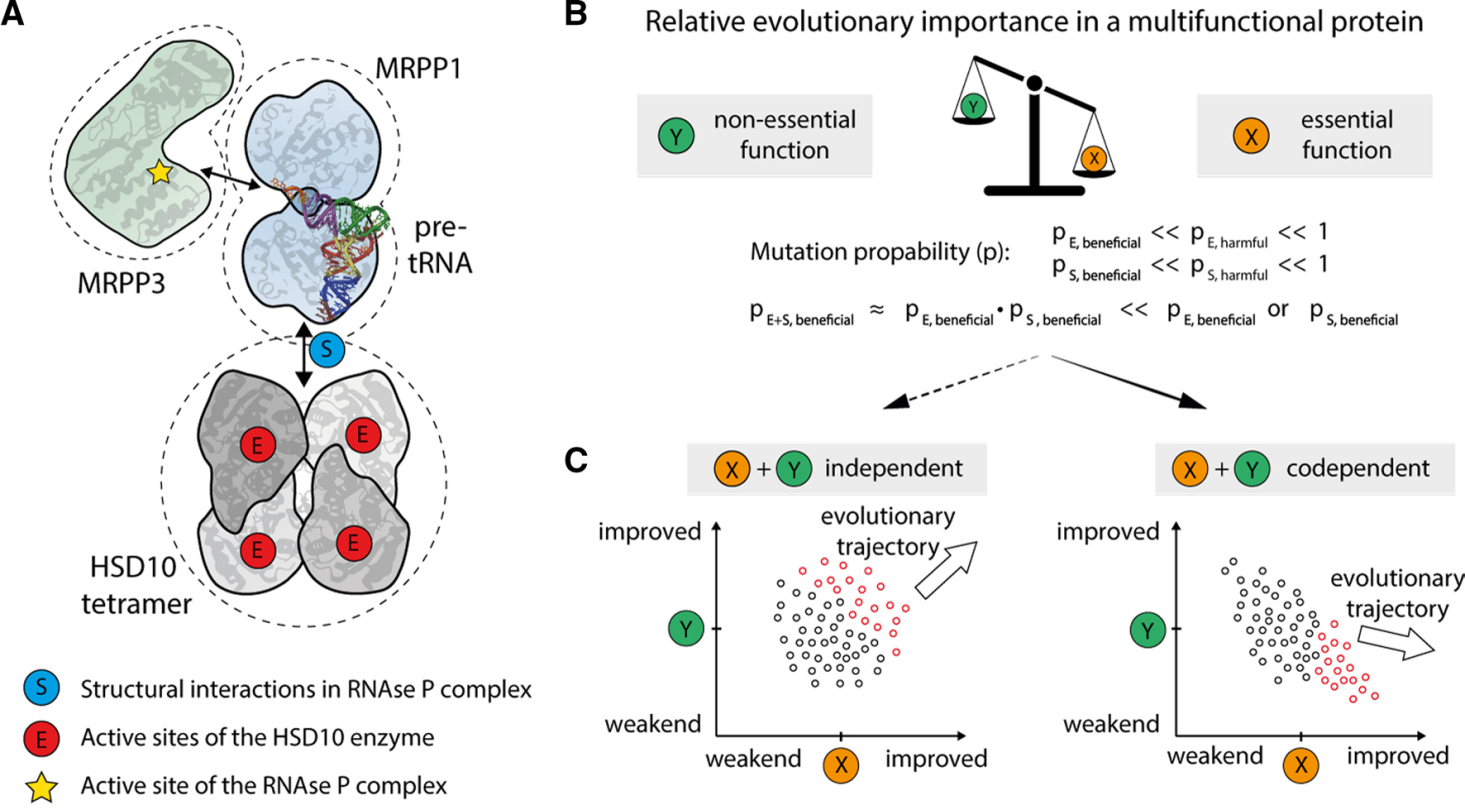
Evolutionary constraints resulting from multifunctional enzymes. **A** The catalytic site of HSD10 (E, red circle) is located at the core of each HSD10 monomer, its structural function in assembling MRPP1, MRPP3, and the pre-tRNA substrate in the mtRNase P complex is mediated by the proteins surface properties (S, blue circles, compare also Fig. 1A). The active site of the mtRNase P complex is located in the MRPP3 subunit (yellow star). **B** In multifunctional proteins an essential function (X, yellow circle) has per definition more evolutionary importance than its non-essential counterpart (Y, green circle). With both functions being determined within the same protein, their evolvability is intertwined. As it is much more likely to acquire a harmful (*P*_E, harmful_ and *P*_S, harmful_) than a beneficial mutation (*P*_E, beneficial_ and *P*_S, beneficial_), it is statistically highly unlikely that any given mutation improves both functions at the same time (*P*_E+S, beneficial_). This results in a negative covariance between the two functions. **C** Left: in a scenario were the essential (X) and non-essential (Y) functions can be evolved independently, a simultaneous improvement of both functions is likely. Right: due to the apparent negative covariance, the selection pressure is much more influenced by the characteristic that provides the greater evolutionary advantage. It follows that an evolutionally improvement in the essential function (X) is preferred, which at the same time can lead to a gradual deterioration in the non-essential function (Y)

It remains surprising that the purified HSD10 enzyme accepts such a broad spectrum of chemically diverse substrates, including CLs (Supplemental Table 1). One possible explanation for this substrate promiscuity arises from the general structural organization of HSD10 and the multifunctional nature of this protein. While the catalytically active site of HSD10 (Fig. 5A, E) is localized inside the protein fold, the amino acid residues relevant for its interactions in the mtRNase P complex (Fig. 5A, S) are found on the surface of the HSD10 tetramer. The substrate binding cavity of HSD10 is exposed to the surface and relatively voluminous (Supplemental Figure S5) in relation to the size of many of its suspected substrates (Supplemental Table 1). In general, a number of evolutionary considerations can be made for a protein that combines an essential and a non-essential function: (1) The essential function will have more evolutionary importance compared to the non-essential function (Fig. 5B). (2) As the vast majority of natural random mutations are either harmful or neutral [50], there remains only a small probability that a mutation is beneficial for one of the two functions (Fig. 5B). (3) If both traits can evolve independently from each other, a simultaneous improvement of both functions is possible (Fig. 5C, left). (4) However, if both functions are co-dependent (e.g. rely on the same structural elements), the chance of one mutation improving both functions at the same time is several orders of magnitude smaller (Fig. 5B), leading to a negative covariance in the accessible evolutionary space (Fig. 5C, right). (5) In this scenario it follows that in an evolutionary selection process, an improvement of the essential function is more impactful, while improvements of the non-essential function are statistically much more likely to be prevented by simultaneous adverse effects on their essential counterpart (Fig. 5C, right). It can be speculated that this model at least partially explains the observed properties of HSD10. In contrast to its enzyme function, the structural role of HSD10 in the mtRNase P complex is essential. As a result, the evolutionary trajectory for HSD10 could have pointed towards a steady improvement of the mtRNase P assembly capacity, which outweighs potential adverse effects on the enzymes substrate specificity. Whether this is the underlying mechanism for the noticeably high number of reported HSD10 substrates in literature has yet to be proven in future experiments.

These potential evolutionary constraints of HSD10 might be relevant for evaluating the properties of other multifunctional proteins as well. The BRENDA database lists a considerable number of enzymes that accept multiple substrates, which is often related to the chemical similarity of the respective compounds [51]. Typically these enzymes evolved to optimize the in vivo specificity for their main substrate [52], although this does not entirely prevent the occurrence of unwanted side reactions [53]. The resulting metabolite damage in several cases requires the action of specialized repair enzymes [53, 54]. Impairment of these repair systems can lead to human disorders [55], which in the ICIMD classification scheme of inborn errors in metabolism are even grouped in a dedicated metabolite repair category [56]. Thus, also when examining the physiological relevance of already known in vitro substrates of HSD10, one should not automatically infer a role that is beneficial for the metabolic network, but also consider the possibility of unwanted metabolite-damaging effects.

The symptoms of HSD10 disease in patients provide further information on the physiological relevance of different enzymatic activities of HSD10. For example, the accumulation of 2-methyl-3-hydroxybutyrate in the patients’ urine particularly after isoleucine challenge confirmed that this enzymatic function in isoleucine catabolism indeed has a physiological contribution to the metabolic network [4, 8, 9]. On the other hand, there is evidence that the core symptoms of HSD10 disease—cardiomyopathy, lactic acidosis, neurodegeneration and mental retardation—develop independently of the protein’s dehydrogenase capability, and are related to the structural function in the mtRNase P complex [4, 8]. Different HSD10 disease-causing variants exhibit residual enzymatic activity in the range of 0–30%, which are, however, not at all correlated with the severity of the disease itself [4, 8]. Furthermore, patients carrying pathogenic variants in the *ELAC2* gene, which encode RNase Z responsible for the 3′ cleavage of mitochondrial RNA, show symptoms comparable with HSD10 disease [57]. In analogy to HSD10 disease, dysfunctional *ELAC2* also leads to the inability of cells to mature mitochondrial tRNAs, leading to the accumulation of precursor mtRNA (as well as a lack of processed mtRNA). However, in this condition all enzymatic functions of HSD10 remain intact, still causing cardiomyopathy, lactic acidosis, and encephalopathy [58], adding to the evidence that the mitochondriopathy in HSD10 disease is caused due to defective mtDNA transcript processing, rather than impaired of mitochondrial membrane lipid homeostasis [57].

In summary, HSD10 is a multifunctional protein that harbours an enzymatic dehydrogenase function with a surprisingly broad range of different substrates in vitro (Supplemental Table 1). The physiological relevance of some of the proposed functions has already been questioned for decades [3]. In the present study, we investigated the previously suggested contribution of HSD10 to CL metabolism and homeostasis (Fig. 1B). Despite testing this hypothesis in patient fibroblasts as well as HSD10-knockdown models in different genetic backgrounds and lipid environments, we were not able to find supporting evidence for such a function (Fig. 1C, 2C, 3B). Biochemical testing of purified HSD10 confirmed that the enzyme can metabolize CLs, but only when the protein is present in excess concentrations, and at slow rates (Fig. 4B). A possible explanation for the in vitro promiscuity of HSD10 could be evolutionary constraints that are caused by two spatially separated but co-dependent functions with unequal physiological importance dictating this protein’s overall evolutionary trajectory (Fig. 4C). This study also demonstrates the importance of experimentally supporting the translation of scientific findings from one conceptual level to another. If there are conditions in which an in vivo CL-cleaving function of HSD10 actually plays a role, these must first be found and proven. Even if this is the case, it would still be important to differentiate if the observed activity is a potentially unwanted metabolite-damaging side reaction or represents a regulated part of CL metabolism.

## Materials and methods

### Cell culture

Cells were cultured, if not stated otherwise, at 37 °C/5% CO_2_ in cell culture medium, consisting of low glucose DMEM (Sigma Aldrich Chemie GmbH, Schnelldorf, Germany) supplemented with 10% fetal bovine serum (FBS) (Gibco, Fisher Scientific GmbH, Vienna, Austria), penicillin/streptomycin (Sigma Aldrich Chemie GmbH), and 2 mM l-glutamine (Lonza, Szabo-Scandic, Vienna, Austria). Medium was exchanged every second day. Cells were pelleted by trypsinization, washing in PBS, and snap-freezing in liquid nitrogen. Pellets were stored at − 80 °C.

### Lipid supplementation

Lipid supplementations were conducted as described [48]. Briefly, cells were supplemented with 25 µM BSA-conjugated palmitic (16:0) and linoleic acid (18:2) (Sigma–Aldrich, St. Louis, USA), respectively. 100 mM fatty acid stock solutions were prepared with 100 mM NaOH, incubated above their melting point and mixed with a 2.8 mM BSA solution (Bovine Serum Albumin; A7030, Sigma–Aldrich, St. Louis, USA). Stock solutions were diluted in cell culture medium and added to the cells.

### Cardiolipin analysis with LC–MS/MS

Lipid extraction and sample analysis were performed as described previously [38, 48], with modifications. Briefly, lipids were extracted from whole cell pellets using the Folch method [59], dried, and subjected to RP-HPLC separation and subsequent time-of-flight mass spectrometric detection. As under most conditions cardiolipins are exclusively present in mitochondria, no additional specific subcellular compartment enrichment strategies are required. Raw data were pre-processed and features were integrated using MZmine [60]. Relative abundance data were obtained via an in-house R-pipeline (http://www.R-project.org). A detailed description is given in Supplemental Text 1 and Supplementary Tables 3–7.

### Knockdown of HSD10 in HEK 293 T cells

Knockdown of *HSD17B10* was performed as described in [61]. Briefly, for the *HSD17B10* knockdown 500.000 wild type HEK 293 T or tafazzin-deficient HEK 293 T cells were cultured in 25 cm^2^ cell culture flasks under standard conditions. 24 h prior to the transfection with 270 nM siRNA targeting the *HSD17B10* gene (targeting sequence: CAGCGAGTTCTTGATGTGAAT, final volume 3 ml) (Qiagen, Hilden, Germany), cells were seeded and maintained at 37 °C/5% CO_2_. AllStars Negative Control siRNA (Qiagen, Hilden, Germany) served as negative control (Control). siRNAs were mixed with Turbofect Transfection Reagent (Thermo Scientific) in a 1:3 ratio, added to the cells and incubated at 37 °C/5% CO_2_ for 48 h. The successful knockdown was confirmed by western blotting.

### Western blot

Western blot analyses were performed under denaturing and reducing conditions as described in [62]. Briefly, 12 µg of protein was separated on a 10% SDS gel (Biorad Laboratories GmbH, California, USA), followed by transfer onto a PVDF membrane via wet blotting and probing with ERAB antibody (ab137455, 1:1000) as primary and polyclonal goat anti rabbit immunoglobulins/HRP (1:4000) as secondary antibody. Bands were detected with ECL (GE Healthcare, Vienna, Austria) and developed on Amersham Hyperfilm (VWR, Vienna, Austria).

### Isoleucine treatment of HSD10 knockdown cells

For detection of 2-methyl-3-OH-butyrate, HSD10 knockdown cells were supplemented with medium containing 1.36 mM isoleucine, which was added 48 h post-transfection from a 5 mM stock solution. Cell culture medium was sampled after 0 h, 24 h, and 48 h for further GC–MS analysis.

### Sample preparation for GCMS analysis

750 µl cell culture media was mixed with 100 µl of 500 µM tricarballylic acid (internal standard) and 100 µl of 5% hydroxylamine acid and incubated at 60 °C for 30 min. 100 µl of 6 M HCl was added, followed by loading on an EXtrelut NT1 column (Merck, Darmstadt, Germany), 10 min of incubation, and elution with 8 ml of 1:1 chloroform/amyl alcohol. Phase separation was achieved by addition of 1 ml 4% ammoniac. The aqueous phase was evaporated at 50 °C under a N_2_ stream, re-dissolved in 500 µl dichloromethane, which was again evaporated. Next, 100 µl of N,O-Bis(trimethylsilyl)trifluoroacetamide containing 1% trimethylchlorosilane was added for derivatization and incubated 1 h at 60 °C [63–65].

### Gas chromatography–mass spectrometry

Sample analysis was performed as described previously [63–65]. Analytes were separated on a GC–MS system equipped with a fused silica capillary column (Perabond SE-54-DF 50 µm thick film, Macherey–Nagel, Oensingen Switzerland). Peaks were analyzed in MzMine [60] with peak integration parameters set to: Intensity tolerance 30%, noise level 1000, *m*/*z* tolerance 100 ppm and retention time tolerance 0.3 min. Integration was manually curated. 2-Methyl-3-OH-butyrate peaks were analyzed at 117 *m*/*z* and RT = 15.4 min and were normalized to the internal standard tricarballyic acid (377 *m*/*z*, RT = 40.45 min).

### Cloning and protein expression of HSD17B10

Cloning and purification of *HSD17B10 *was conducted in analogy to 40. *HSD17B10* lacking the MTS sequence (aa 1–11) was cloned from human complementary DNA using ligation-independent cloning-(LIC)-compatible PCR primers (MRPP2_∆11_F:TTTAAGAAGGAGATATAGATCATGGTGGCGGTAATAACCGGAGGAGCC; MRPP2_∆11_R:TTATGGAGTTGGGATCTTATTAAGGCTGCATACGAATGGCCCCATCC). The PCR products were purified (QIAquick gel extraction kit, Qiagen) and cloned into the pET-derived 14-A vector (Addgene, #48307, kind gift of S. Gradia). A 6XHis purification tag and the MTS sequence (aa 1–11) were then added to the N-terminus using around-the-horn PCR. For this, phosphorylated primers (6XHis_MRPP2_F:GCAGCAGCGTGTCGGAGCGTGAAGGGCCTGGTGGCGGTAATAACCGGAG;6XHis_MRPP2_R:ATGGTGATGGTGATGGTGCATGATCTATATCTCCTTCTTAAAGCTTAAAG) were used to amplify the entire vector and the amplicon gel purified, ligated and transformed in E.coli XL-1 Blue to generate the 14A-6XHis-*HSD17B10* expression construct.

6XHis-*HSD17B10* was expressed in E. coli BL21 (DE3) RIL cultured in LB media supplemented with 100 µg/mL of ampicillin and 34 µg/mL of chloramphenicol. The expression culture was grown at 37 °C, 120 rpm until it reached an optical density of 0.5 at 600 nm, followed by 30 min incubation at 16 °C. Expression was induced with 150 µM isopropyl β-d-1-thiogalactopyranoside at 16 °C for 18 h. The cells were harvested through centrifugation and the cell pellets snap-frozen with liquid nitrogen and stored at − 80 °C.

### 6XHis-HSD17B10 purification

All purification steps were carried out at 4 °C. The cell pellet was resuspended in lysis buffer (50 mM HEPES pH 7.5 at 4 °C, 300 mM NaCl, 30 mM Imidazole, 2 mM DTT, 10% glycerol) supplemented with cOmpleteTM protease inhibitor cocktail (Roche) and lysed by sonication. The lysate was centrifuged twice at 47,807*g* for 30 min each to remove cell debris. The supernatant was collected and loaded into a HisTrap HP 5 mL column (Cytiva) equilibrated with lysis buffer. The column was washed with 5 CV of lysis buffer followed by 9.5 CV of high salt buffer (50 mM HEPES pH 7.5 at 4 °C, 1 M NaCl, 30 mM Imidazole, 2 mM DTT, 10% glycerol) and finally 5 CV of lysis buffer. The column was eluted with elution buffer (35 mM HEPES pH 7.5 at 4 °C, 210 mM NaCl, 621 mM Imidazole, 1.4 mM DTT, 7% glycerol) and fractions containing 6XHis-HSD17B10 were pooled. The eluate was dialysed against dialysis buffer (50 mM HEPES pH 7.5 at 4 °C, 100 mM NaCl, 2 mM DTT, 10% glycerol) first for two hours and then again for 16 h. The dialyzed eluate was then loaded onto a HiTrap Heparin HP 5 mL column (Cytiva) equilibrated with 95% (v/v) of heparin affinity chromatography (HAC) buffer A (50 mM HEPES pH 7.5 at 4 °C, 10% glycerol, 2 mM DTT) and 5% (v/v) HAC buffer B (50 mM HEPES pH 7.5 at 4 °C, 2 M NaCl, 2 mM DTT, 10% glycerol). The column was washed with the same buffer mixture and the proteins were eluted with a linear gradient of 5–100% (v/v) of HAC buffer B in HAC buffer A. Fractions containing *HSD17B10* were pooled and concentrated using an Amicon^®^ Ultra-15 10 K MWCO concentrator (Merck) and the protein was further purified by size exclusion chromatography using a Superdex 200 Increase 10/300 GL column (Cytiva) equilibrated in SEC buffer (20 mM HEPES pH 7.5 at 4 °C, 150 mM NaCl, 5 mM DTT, 5% glycerol). Elution fractions containing *HSD17B10* were collected and concentrated using an Amicon^®^ Ultra-4 10 K MWCO concentrator (Merck) to a final concentration of 5.4 mg/ml. Aliquots of *HSD17B10* were flash frozen in liquid nitrogen and stored at – 80 °C until use.

### Measurement of HSD10 enzyme activity

Kinetic constants of HSD10 wild type and mutants were determined with a FLUOStar Omega Microplate Reader (BMG LABTECH, Ortenberg, Germany) via the conversion of the cofactor NAD^+^ to NADH, which was followed fluorometrically (excitation: 340–10 nm; emission: 460 nm). Reactions were carried out in 96-well plates in a assay buffer at a pH of 8.0, with final concentrations of 10 mM Tris/HCl, 1 mM EDTA, 0.1% (w/v) sodium cholate, and 937 µM NAD^+^, modified from [11]. 3-Hydroxybutyryl-CoA (H0261, Sigma Aldrich), CL(18:1)_4_ (710335P, Sigma Aldrich) and cardiolipin mix from bovine heart (C0563, Sigma Aldrich) were used as substrates in the range of 0–600 μM and added to the assay buffer. 3-hydroxybutyryl-CoA was dissolved in ddH_2_O and CLs were dissolved in MeOH. The amount of MeOH was controlled for in the final assay mix. 1.8 mg/ml of purified HSD10 were diluted 1:30 to 1:3000 in Tris/HCl pH 8.0 in the presence of 500 µM NAD^+^. Reactions were started by addition of 100 µl protein solution to 60 µl assay buffer and was kinetically followed for up to 60 min. The obtained fluorescence data were analyzed with the MARS software (BMG LABTECH). Reaction rates were determined via the slope within the linear range of the resulting curves and calibrated with an external NADH dilution series.

## Electronic supplementary material

Below is the link to the electronic supplementary material.

Supplementary file1 (PDF 904 KB)

## Data Availability

The mass spectrometry data results have been made available on Mendeley Data (10.17632/k4j5kjfj7t.1).
