# Supplementary material for: Lost in promiscuity? An evolutionary and biochemical evaluation of HSD10 function in cardiolipin metabolism
Source: Cell Mol Life Sci. 2022 Oct 22;79(11):562. doi: 10.1007/s00018-022-04579-6 (PMC9587951; doi:10.1007/s00018-022-04579-6)
Supplement: Supplementary file 1 — Supplementary file1 (PDF 904 KB) [file 18_2022_4579_MOESM1_ESM.pdf]

Supplement

Supplemental Table 1: List of enzyme activities attributed to HSD10

Metabolic pathway	Substrate	Product	Reference
Organic acids	3-Hydroxy-2-Methylbutyryl-CoA	2-Methyl-3-Oxobutanoyl-CoA	[1]
	3-Hydroxybutyrate	Acetoacetat	[2]
Lipid metabolism	Cardiolipin	DAG, DHA and Pi*	[3]
	Acetoacetyl-CoA	L-3-Hydroxybutyryl CoA	[4, 5]
	3-Ketooctanoyl-CoA	3-Hydroxy- Octanoyl-CoA	[4, 5]
Alcohol	Butanol	Butyl aldehyde	[2]
	n-Decanol	Decanal	[6]
	2-Propanol (secondary alcohols)	Acetone	[4, 5]
Steroid metabolism	17β-Estradiol	Estrone	[2, 5, 7, 8]
	5α-Dihydrosterone	Dihydroandrosterone	[5]
	Androsterone	Androstenedionemoon	[5, 8, 9]
	3α -Androstanediol	5α-Dihydrotestosterone	[7–10]
	3α,5α-3,21-Dihydroxypregnan-20-one	5α-Dihydrodeoxycorticosterone	[11]
	Allopregnanolone (3,5-tetrahydroprogesterone)	5α-Dihydroprogesterone	[12]
	3α,5α- Allotetrahydrodeoxycorticosterone	5α-Dihydrodeoxycorticosterone	[12]
	Androstanedione	Androsterone	[8]

Supplemental Table 2: Patient mutations

Deduced effect on protein	Mutation	Clinical presentation (general)	Sex	Reference
p.A154T	c.460G>A	Milder than juvenile form	M	[13]
p.R130C	c.388C>T	Infantile	M	[1]
p.R226Q	c.677G>A	Neonatal	M	[14]
p.Q165H	c.495A>C	Atypical presentation	M	[15]

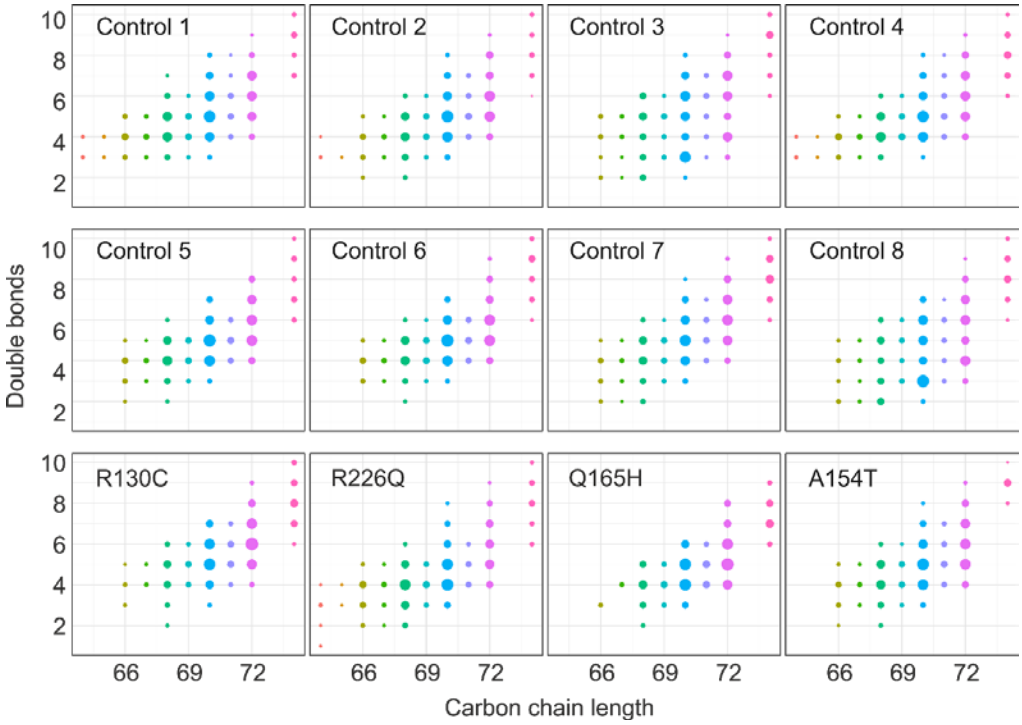

Supplemental Figure S1: Relative single CL-profiles with their respective carbon chain length and double bond amount, whereas the dot size corresponds to the relative abundance of CLs. Plot was normalized to 1, which represents the total amount of CLs. Comparison between fibroblasts obtained from unaffected healthy controls (Controls) and fibroblasts derived from HSD10 disease patients (Mutation indicated).

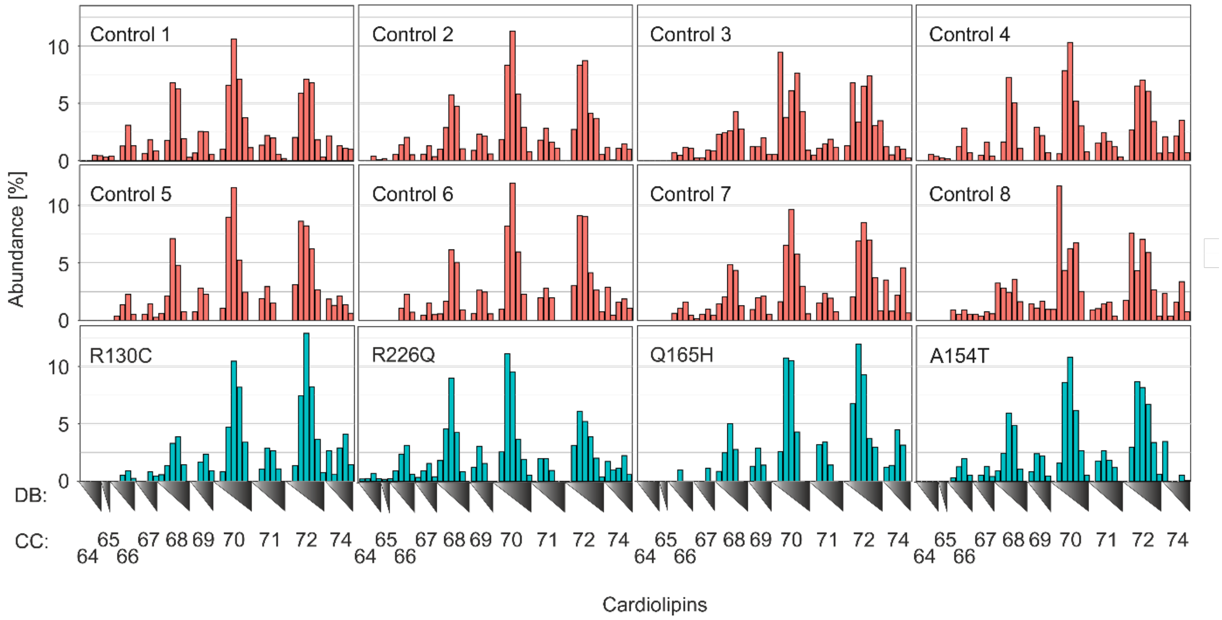

Supplemental Figure S2: Relative single CL-profiles of patient fibroblasts (turquoise) and healthy controls (red). Profiles are shown as mean ± SD and are normalized to total CL. CL species are indicated by their total number of side chain carbon atoms (CC) and sorted according to the number of double bonds (DB)

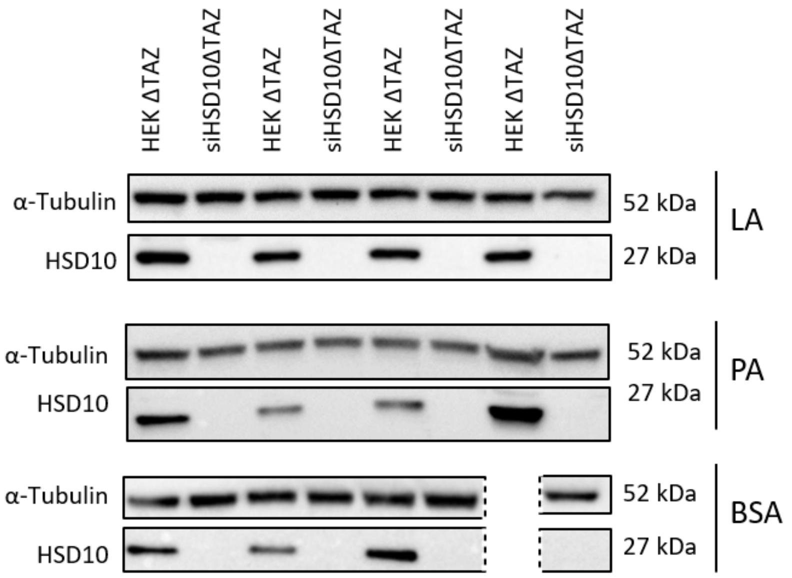

Supplemental Figure S3: Conformation of HSD10 knockdowns (siHSD10) in tafazzin deficient HEK 293T cells (HEKΔTAZ) via western blot analysis. α-Tubulin was used as loading control. HSD10 was detected with the ERAB-antibody (ab137455). Additionally, cells were supplemented with BSA as control, with 25 µM palmitic acid (PA) and with 25 µM linoleic acid (LA) for 72h.

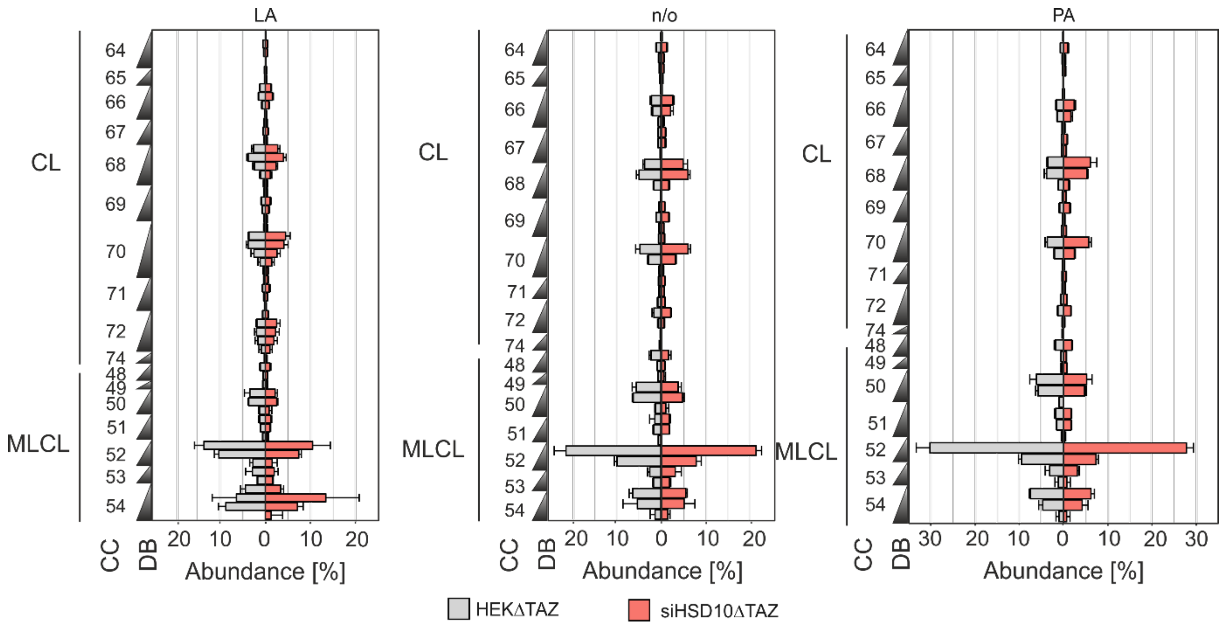

Supplemental Figure S4: Comparison between tafazzin deficient HEK control cells (HEKΔTAZ) and HSD10 knockdown cells (siHSD10ΔTAZ). Cells were supplemented with linoleic (LA (18:2)) and palmitic acid (PA (16:0)) to create a polyunsaturated and saturated environment. CL species are indicated by their total number of side chain carbon atoms (CC) and sorted according to the number of double bonds (DB)

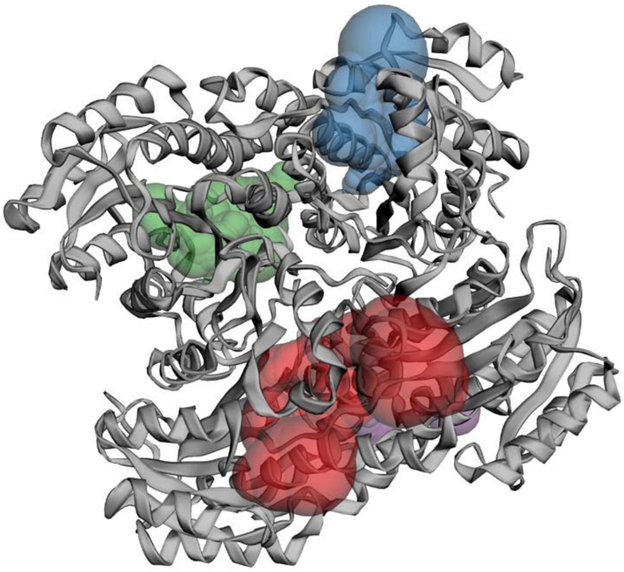

Supplemental Figure S5: HSD10 tetramer (PDB code: 1U7T) displayed with the four substrate binding cavities (subunit A: red, subunit B: blue, subunit C: green, subunit D: purple). The cavity volume was modelled and illustrated employing the Castp 3.0 Server, using a 1.4 Å probe radius (PMID: 29860391).

Supplemental Text 1

Lipid extraction and detection by LC-MS/MS

Lipid extraction from cell and fibroblast pellets was performed according to Folch [16]. Briefly, samples were homogenized and lipids were extracted with a 2/1 Chloroform/MeOH extraction solvent, containing 0.5 µM internal standards ($CL(14:0)_4$, $PE(14:0)_2$, $PC(14:0)_2$, $PG(14:0)_2$, $PI(14:0)_2$, $PA(14:0)_2$). Prior to HPLC-MS/MS measurements extracts were dissolved in salt-free starting solvent and loaded into the autosampler.

Experiments were performed on a trapped ion mobility spectrometry (tIMS) time-of-flght (TOF) mass spectrometer coupled to a Bruker Elute uHPLC (Bruker Daltonics, Bremen Germany) combined with an Agilent Poroshell 120 EC-C8 2.7mm 2.1x100mm column (Agilent Technologies, Santa Clara, USA). The HPLC setup is summarized in Supplemental Table 3. The HPLC method was adapted from [17, 18].

Supplemental Table 3: HPLC-Setup

Column	Agilent Poroshell 120 EC-C8 2.7mm 2.1x100mm column
Column oven temperature	40°C
Autosampler temperature	10°C
Solvent A	10 mM ammonium formate (for LC-MS, VWR, Pensylvania, Radnor) and 0.2% formic acid (for LC-MS, VWR, Pensylvania, Radnor) in 60/40 Acetonitrile/Water
Solvent B	10 mM ammonium formate (for LC-MS, VWR, Pensylvania, Radnor) and 0.2% formic acid (for LC-MS, VWR, Pensylvania, Radnor) in 90/10 Isopropanol/Acetonitrile
Injection Volume	20 µl for fibroblasts / 10µl for cell culture cells
Injection mode	µl pick up mode

Sample was injected onto an Agilent Poroshell 120 EC-C8 2.7mm 2.1x100mm column at 40°C with column guard (Agilent Technologies, Santa Clara, USA) with a flow rate of 0.4 ml/min. Starting with 60% of solvent B and 40% of solvent A an isocratic elution was performed for 1 minute, continued with a gradient increasing to 70% B within 14 minutes. This was followed by a wash phase and a 2 min reequilibration phase at 60 % B. The total runtime was 24 min.

The mass spectrometer setup is summarized in Supplemental Table 4. The mass spectrometric method was adapted from [17, 18].

Supplemental Table 4: MS-Setup with tuning parameters

Full MS mass range	400-1750 m/z
Spectra Rate	5 Hz
End Plate Offset	500 V
Nebulizer	2.5 Bar
Dry Temperature	250°C
Capillary	4500 V
Dry Gas	10.0 l/min
**Tuning Parameters:**	
Funnel 1 RF	350.0 Vpp
Deflection Delta	-90.0 V
Multipole RF	500.0 Vpp
Collision RF	1100.0 Vpp
isCID Energy	0.0 eV
Funnel 2 RF	550.0 Vpp
Ion Energy	10.0 eV
Collision Energy	20.0 eV
Transfer Time	70.0 µs

The mass spectrometric data acquisition was performed with otofControl (version 6.2.1.2, Bruker Daltonics, Bremen, Germany) in ESI-negative mode. The mass range from 400-1750 m/z was monitored with a spectra rate of 5 Hz, recording line and profile spectra with a maximum intensity peak detection. Overall source parameters are shown in Supplemental Table 4. The raw data processing parameters were set as listed in Supplemental Table 5:

Supplemental Table 5: General processing parameters

Processing Classic	On
Peak Summation Width	5 counts
Use Max. Intensity	On
Calculate Peak Width	On
Calculate Quality Factor	Off
Guessed Noise	200
Average Noise	1
Flatness	0.14
Guessed Average	100

Acquisition of cardiolipin analyte data was performed with line spectra thresholds of 1000 counts for cell culture and 100 counts for fibroblast samples with a lower mass cutoff of 950 m/z and a pre pulse storage time of 25 µs. Mass calibration was achieved in a separate calibration segment with divert valve injection of 20 µL of a 5 mM sodium formate solution. MS^2 acquisition was performed in auto MS/MS mode (Supplemental Tables 6 and 7):

Supplemental Table 6: Auto MS/MS mode

Precursor Ion List	Exclude
MS Repetitions	1x
Threshold (per 1000 sum.) Absolute	100 counts
Exclude after	1 Spectra
Reconsider Precursor	On
Smart Exclusion	5x
No. of Precursors	1x
MS/MS Repetitions	1x
Active Exclusion	On
Release after	0.05 min
If Current Intensity/Previous Intensity	5.0
Smart Exclusion	5x

Supplemental Table 7: MS/MS preferences

Preferred Range	Off
Preferred Range Low	1
Exclude Singly	Off
Group Length	5
Sort Precursors by	Intensity
Preferred Range High	1
Exclude unknown	Off
Strict Active Exclusion	Off
CID Fallback Charge State	1 z
Isolation and Fragmentation List	400-1000: 45 eV,<br>1001-1700: 65 eV,<br>Exclusion width: 1 m/z, Charge state: 1

Lipidomic data analysis

Data was converted into the open .mzML format, including a centroiding step using the MSconvert GUI (Version: 3.0.21037) and then was analysed in Mzmine (Version 2.53) [19] with the targeted feature extraction method. Raw peak areas for monoisotopic and the first isotope peak of relevant features were exported and further processed with our in-house pipeline in R (Version 4.1.2.).

References:

1. Zschocke J, Ruiter JPN, Brand J, et al (2000) Progressive Infantile Neurodegeneration Caused by 2-Methyl-3-Hydroxybutyryl-CoA Dehydrogenase Deficiency: A Novel Inborn Error of Branched-Chain Fatty Acid and Isoleucine Metabolism. Pediatr Res 48:852–855. https://doi.org/10.1203/00006450-200012000-00025

2. Powell A., Read J., Banfield M., et al (2000) Recognition of structurally diverse substrates by type II 3-hydroxyacyl-CoA dehydrogenase (HADH II)/Amyloid-β binding alcohol dehydrogenase (ABAD). J Mol Biol 303:311–327. https://doi.org/10.1006/jmbi.2000.4139

3. Boynton TO, Shimkets LJ (2015) Myxococcus CsgA, Drosophila Sniffer, and human HSD10 are cardiolipin phospholipases. Genes Dev 29:1903–1914. https://doi.org/10.1101/gad.268482.115

4. He X-Y, Schulz H, Yang S-Y (1998) A Human Brain l-3-Hydroxyacyl-coenzyme A Dehydrogenase Is Identical to an Amyloid β-Peptide-binding Protein Involved in Alzheimer’s Disease. J Biol Chem 273:10741–10746. https://doi.org/10.1074/jbc.273.17.10741

5. He X-Y, Merz G, Mehta P, et al (1999) Human Brain Short Chain l-3-Hydroxyacyl Coenzyme A Dehydrogenase Is a Single-domain Multifunctional Enzyme. J Biol Chem 274:15014–15019. https://doi.org/10.1074/jbc.274.21.15014

6. Yan S Du, Shi Y, Zhu A, et al (1999) Role of ERAB/L-3-hydroxyacyl-coenzyme A dehydrogenase type II activity in Aβ-induced cytotoxicity. J Biol Chem 274:2145–2156. https://doi.org/10.1074/jbc.274.4.2145

7. Shafqat N, Marschall H-U, Filling C, et al (2003) Expanded substrate screenings of human and Drosophila type 10 17β-hydroxysteroid dehydrogenases (HSDs) reveal

multiple specificities in bile acid and steroid hormone metabolism: characterization of multifunctional 3α/7α/7β/17β/20β/21-HSD. Biochem J 376:49–60. https://doi.org/10.1042/bj20030877

8. He X-Y, Merz G, Yang Y-Z, et al (2000) Function of human brain short chain L-3-hydroxyacyl coenzyme A dehydrogenase in androgen metabolism. Biochim Biophys Acta - Mol Cell Biol Lipids 1484:267–277. https://doi.org/10.1016/S1388-1981(00)00014-7

9. He X-Y, Yang Y-Z, Schulz H, Yang S-Y (2000) Intrinsic alcohol dehydrogenase and hydroxysteroid dehydrogenase activities of human mitochondrial short-chain L-3-hydroxyacyl-CoA dehydrogenase. Biochem J 345:139. https://doi.org/10.1042/0264-6021:3450139

10. Yang S-Y, He X-Y, Olpin SE, et al (2009) Mental retardation linked to mutations in the HSD17B10 gene interfering with neurosteroid and isoleucine metabolism. Proc Natl Acad Sci 106:14820–14824. https://doi.org/10.1073/pnas.0902377106

11. Belelli D, Lambert JJ (2005) Neurosteroids: endogenous regulators of the GABAA receptor. Nat Rev Neurosci 6:565–575. https://doi.org/10.1038/nrn1703

12. He X-Y, Wegiel J, Yang Y-Z, et al (2005) Type 10 17beta-hydroxysteroid dehydrogenase catalyzing the oxidation of steroid modulators of γ-aminobutyric acid type A receptors. Mol Cell Endocrinol 229:111–117. https://doi.org/10.1016/j.mce.2004.08.011

13. Fukao T, Akiba K, Goto M, et al (2014) The first case in Asia of 2-methyl-3-hydroxybutyryl-CoA dehydrogenase deficiency (HSD10 disease) with atypical presentation. J Hum Genet 59:609–614. https://doi.org/10.1038/jhg.2014.79

14. García-Villoria J, Navarro-Sastre A, Fons C, et al (2009) Study of patients and carriers with 2-methyl-3-hydroxybutyryl-CoA dehydrogenase (MHBD) deficiency: Difficulties in the diagnosis. Clin Biochem 42:27–33. https://doi.org/10.1016/j.clinbiochem.2008.10.006

15. Rauschenberger K, Schöler K, Sass JO, et al (2010) A non-enzymatic function of 17β-hydroxysteroid dehydrogenase type 10 is required for mitochondrial integrity and cell survival. EMBO Mol Med 2:51–62. https://doi.org/10.1002/emmm.200900055

16. Folch J, Lees M, Stanley GHS (1957) A simple method for the isolation and

purification of total lipides from animal tissues. J Biol Chem 226:497–509. https://doi.org/10.1016/S0021-9258(18)64849-5

17. Oemer G, Lackner K, Muigg K, et al (2018) Molecular structural diversity of mitochondrial cardiolipins. Proc Natl Acad Sci 115:4158–4163. https://doi.org/10.1073/pnas.1719407115

18. Oemer G, Koch J, Wohlfarter Y, et al (2020) Phospholipid Acyl Chain Diversity Controls the Tissue-Specific Assembly of Mitochondrial Cardiolipins. Cell Rep 30:4281-4291.e4. https://doi.org/10.1016/j.celrep.2020.02.115

19. Pluskal T, Castillo S, Villar-Briones A, Orešič M (2010) MZmine 2: Modular framework for processing, visualizing, and analyzing mass spectrometry-based molecular profile data. BMC Bioinformatics 11:395. https://doi.org/10.1186/1471-2105-11-395
